# Incidence and Prognostic Factors of Radial Artery Occlusion in Transradial Coronary Catheterization

**DOI:** 10.3390/jcm13113276

**Published:** 2024-06-01

**Authors:** Matthaios Didagelos, Areti Pagiantza, Andreas S. Papazoglou, Dimitrios V. Moysidis, Dimitrios Petroglou, Stylianos Daios, Vasileios Anastasiou, Konstantinos C. Theodoropoulos, Antonios Kouparanis, Thomas Zegkos, Vasileios Kamperidis, George Kassimis, Antonios Ziakas

**Affiliations:** 1 1st Cardiology Department, AHEPA University General Hospital, 54636 Thessaloniki, Greece; apayantza@gmail.com (A.P.); stylianoschrys.daios@gmail.com (S.D.); vasianas44@gmail.com (V.A.); ktheod2005@hotmail.com (K.C.T.); akouparanis@gmail.com (A.K.); zegkosth@gmail.com (T.Z.); vkamperidis@outlook.com (V.K.); tonyziakas@hotmail.com (A.Z.); 2 424 General Military Hospital, 56429 Thessaloniki, Greece; dimoysidis@gmail.com (D.V.M.); dimpetroglou@yahoo.gr (D.P.); 3 Athens Naval Hospital, 11521 Athens, Greece; anpapazoglou@yahoo.com; 4 2nd Department of Cardiology, Hippokration Hospital, 54942 Thessaloniki, Greece; gksup@yahoo.gr

**Keywords:** transradial access (TRA), radial artery occlusion (RAO), percutaneous coronary intervention (PCI), coronary artery disease (CAD)

## Abstract

**Background/Objectives**: Radial artery occlusion (RAO) is the most common complication of transradial coronary catheterization. In this study, we aimed to evaluate the incidence of RAO and identify the risk factors that predispose patients to it. **Methods**: We conducted an investigator-initiated, prospective, multicenter, open-label study involving 1357 patients who underwent cardiac catheterization via the transradial route for angiography and/or a percutaneous coronary intervention (PCI). Univariate and multivariate logistic regression analyses were performed to identify potential predictors of RAO occurrence. Additionally, a subgroup analysis only for patients undergoing PCIs was performed. **Results**: The incidence of RAO was 9.5% overall, 10.6% in the angiography-only group and 6.2% in the PCI group. Independent predictors of RAO were as follows: (i) the female gender (aOR = 1.72 (1.05–2.83)), (ii) access site cross-over (aOR = 4.33 (1.02–18.39)), (iii) increased total time of the sheath in the artery (aOR = 1.01 (1.00–1.02)), (iv) radial artery spasms (aOR = 2.47 (1.40–4.36)), (v) the presence of a hematoma (aOR = 2.28 (1.28–4.06)), (vi) post-catheterization dabigatran use (aOR = 5.15 (1.29–20.55)), (vii) manual hemostasis (aOR = 1.94 (1.01–3.72)) and (viii) numbness at radial artery ultrasound (aOR = 8.25 (1.70–40)). Contrariwise, two variables were independently associated with increased odds for radial artery patency (RAP): (i) PCI performance (aOR = 0.19 (0.06–0.63)), and (ii) a higher dosage of intravenous heparin per patient weight (aOR = 0.98 (0.96–0.99)), particularly, a dosage of >50 IU/kg (aOR = 0.56 (0.31–1.00)). In the PCI subgroup, independent predictors of RAO were as follows: (i) radial artery spasms (aOR = 4.48 (1.42–14.16)), (ii) the use of intra-arterial nitroglycerin as a vasodilator (aOR = 7.40 (1.67–32.79)) and (iii) the presence of symptoms at echo (aOR = 3.80 (1.46–9.87)), either pain (aOR = 2.93 (1.05–8.15)) or numbness (aOR = 4.66 (1.17–18.57)). On the other hand, the use of intra-arterial verapamil as a vasodilator (aOR = 0.17 (0.04–0.76)) was independently associated with a greater frequency of RAP. **Conclusions**: The incidence of RAO in an unselected, all-comers European population after transradial coronary catheterization for angiography and/or PCIs is similar to that reported in the international literature. Several RAO prognostic factors have been confirmed, and new ones are described. The female gender, radial artery trauma and manual hemostasis are the strongest predictors of RAO. Our results could help in the future identification of patients at higher risk of RAO, for whom less invasive diagnostic procedures maybe preferred, if possible.

## 1. Introduction

Transradial access is nowadays strongly preferred as the default access route for coronary procedures, coronary angiography and/or percutaneous coronary interventions (PCIs) [1,2,3,4,5,6,7,8]. Nevertheless, it is widely acknowledged that its utilization is not devoid of challenges and complications, such as radial artery occlusion (RAO), arterial spasms, the perforation of the artery, forearm hematomas, bleeding at the puncture site, pseudoaneurysm formation, arterio-venous fistulas, nerve injury, sterile granulomas, eversion endarterectomies and skin necrosis [9,10,11,12].

RAO is the most common complication of transradial catheterization, with a varying overall rate ranging from 0.8 to 38% (with a mean incidence of about 5–10%) in the published literature. This variability can be explained by differences in the timing of evaluations; the methods used for diagnosis (pulse palpation vs. ultrasound) and the periprocedural anticoagulation; and the evolution of the puncture technique (ultrasound-guided), the equipment (lower-profile sheaths and catheters) and hemostatic protocols (patent hemostasis, the simultaneous pressure of both radial and ulnar arteries during hemostasis) [13,14,15,16,17,18,19,20,21]. Moreover, RAO incidence differs when evaluated after a diagnostic coronary angiography only (5.9–8.8%) versus after a PCI (4.0–4.5%) [Rashid2016, Hahalis2017].

Although usually considered asymptomatic, RAO may be symptomatic in up to 31–58.8% of patients, presenting as pain in the forearm, numbness/paresthesia, paresis or acute ischemia [20,22,23,24,25,26]. Additionally, in case of RAO, the affected radial artery is excluded from (i) future catheterization procedures, (ii) hemodialysis shunts, (iii) invasive hemodynamic monitoring and (iv) coronary arterial bypass grafting [11,27,28,29,30]. So, it is widely accepted nowadays that the prevention of RAO is of clinical importance and should be carefully considered in transradial catheterization procedures.

The evaluation of the radial artery’s patency after a transradial procedure can be accomplished clinically by the pulse palpation of the artery (the easiest method, though it is not recommended because of the significant underestimation of RAO), the reverse Allen test and the reverse Barbeau test (plethysmography). The gold standard diagnostic method is the radial artery ultrasound, which enables the evaluation of the blood flow in the radial artery, detection of any damage to the arterial wall, measurement of the artery’s diameter and diagnosis of any other complication. The most recent, more sophisticated and operator-independent diagnostic method that can evaluate radial artery perfusion following transradial catheterization is laser perfusion imaging (LPI), although it is not widely available and requires more specialized hardware and software than ultrasound [11,21,28,30,31,32,33,34,35,36].

Regarding pathophysiology, RAO is considered a thrombotic phenomenon due to endothelial injury and decreased blood flow in the radial artery from the puncture, sheath insertion and catheter manipulation. Moreover, intimal hyperplasia, as a result of repeated ipsilateral transradial access and blood stasis due to radial artery compression during hemostasis, provides a favorable environment for thrombus formation and subsequent RAO. Histopathological examinations of specimens aspirated from occluded radial arteries and imaging modalities (vascular ultrasound, angiography, optical coherence tomography) support the theory that thrombus formation is the main cause of RAO [25,28,37,38,39,40,41,42,43].

Risk factors that predispose patients to RAO that have been identified in the literature include age, the female gender, a low body mass index (BMI), diabetes mellitus, chronic kidney disease, peripheral arterial disease, smoking, a previous ipsilateral radial artery puncture, inadequate periprocedural anticoagulation, the administration and type of intra-arterial vasodilators (sheath size, radial artery diameter and a sheath-to-radial artery diameter ratio > 1), repeated radial punctures, radial artery spasms, aspirin administration, and the duration and technique of hemostasis. These can be divided into pre-procedural (non-modifiable), procedural (modifiable) and post-procedural (also modifiable). Several studies have tried to accurately identify them and describe methods, techniques and materials to overcome them in order to reduce RAO rates; however, there is conflicting evidence for some of them [11,16,21,30,44].

The aim of this study was to record the incidence of RAO in a European population, investigate the aforementioned predisposing risk factors of RAO, and evaluate new predictors of this complication in a real-world cohort of more than 1000 patients undergoing transradial catheterization procedures.

## 2. Materials and Methods

### 2.1. Study Design and Setting

We conducted an investigator-initiated, prospective, multicenter, open-label trial to evaluate the incidence and predictors of RAO in patients who underwent a coronary catheterization procedure through transradial access at five tertiary hospitals’ cardiac catheterization labs in Greece.

### 2.2. Patient Selection

Adult patients of both genders who underwent a coronary catheterization procedure (angiography and/or PCI) through conventional transradial access in the forearm (proximal radial) for any indication and who successfully received at least one radial artery sheath of any size at the end of the procedure (irrespective of the outcome of the procedure) were included in this study. Exclusion criteria were as follows: puncture of any other artery at the ipsilateral arm for initial access (brachial, ulnar, or distal radial) or any previous arterial puncture in the ipsilateral arm (brachial, radial, ulnar, or distal radial), cardiogenic shock, death during the procedure, anemia (hemoglobin < 9 g/dL), thrombocytopenia (platelet count < 100,000/μL), dialysis, inability to provide written consent and inability to perform radial artery ultrasound at 24 h.

### 2.3. Data Collection

A variety of variables, including patients’ demographics, medical history, medication, laboratory exams, reason for catheterization, details about the catheterization procedure (radial sheath size, total heparin administered, undergoing PCI or not, and type and duration of local hemostasis), symptoms, bleeding or other access site complications, were recorded and analyzed. Local forearm hematomas were described according to the Early Discharge after Transradial Stenting of Coronary Arteries (EASY) classification [Bertrand2006]. Bleeding events were categorized according to the BARC definition [45].

RAO was detected by radial artery ultrasound at 24 h after the procedure. Any forearm symptoms/signs (pain, numbness, paresis/paralysis, or acute ischemia) were also recorded.

### 2.4. Peri-Procedural Characteristics and Evaluation of Radial Artery Patency

Pre-procedurally, no clinical test (Allen, Barbeau) or ultrasound was performed to evaluate forearm arteries and collateral circulation of the palmar arch.

Radial artery puncture was performed at the forearm, 1–2 cm proximal to the styloid process of the radius, following universally standardized protocols. All patients received intra-arterial vasodilators (either 200 μg nitroglycerin or 2.5 mg verapamil, but not both) and 50–100 IU/kg intravenous (IV) unfractionated heparin (UFH) depending on attending physician’s discretion. Sheath and catheter size was also decided by each operator. No slender sheaths or sheathless catheters were used. Hemostasis was applied either by a radial artery compression device (wrist bracelet with air-filled compression balloon) or by manual compression, trying to achieve patent hemostasis when possible.

Radial artery ultrasound was used to evaluate radial artery patency after successful hemostasis 24 h after the procedure. RAO was defined as complete loss of antegrade blood flow at ultrasound. Radial artery diameter at the puncture site was also measured during the ultrasound examination (inner-to-inner wall). Post-procedurally, radial artery patency was also evaluated clinically as follows: 1. Radial artery pulse palpation; 2. Reverse modified Allen test; and 3. Reverse Barbeau test (oximetry/plethysmography test). Successful hemostasis was defined as no forearm hematoma or bleeding during the first 24 h after the procedure.

### 2.5. Statistics

Baseline study characteristics were compared using the χ2 test for categorical variables and the non-parametric Mann–Whitney U test for continuous variables. The latter test was employed for the comparison of continuous data since they did not follow normal distribution as suggested through the Kolmogorov–Smirnov normality test. Continuous variables are presented as median with interquartile range (IQR), and categorical variables are presented as frequencies with percentages (%). Univariate logistic regression analyses were performed to identify potential predictors of RAO occurrence yielding unadjusted odds ratios (ORs) with 95% confidence intervals (CIs). Variables with univariate *p*-value ≤ 0.10 and variables of clinical relevance (with less than 25% missing values) were forced into a multivariate logistic regression model based on the rule of thumb of a minimum of 10 RAOs per variable to investigate whether there are any independent predictors of RAO occurrence. Additionally, we performed a subgroup analysis only for patients undergoing PCIs, in which only patient gender and dose of intravenous heparin were included as covariates for multivariate adjustment, given the limited number of RAOs in this subgroup population. Data analyses were performed using SPSS 27 (IBM Corp., Armonk, NY, USA), and statistical significance was set at *p* ≤ 0.05.

### 2.6. Study Approval and Ethics

The study was conducted in accordance with the principles set by the Declaration of Helsinki, the International Conference on Harmonization Guidelines for Good Clinical Practice and all applicable regulatory requirements. The study protocol was approved by the Scientific Committee of the AHEPA University General Hospital of Thessaloniki (protocol number: 79/9.2.2018). Informed written consent was provided by each patient before participating in the study.

## 3. Results

Our study included 1357 patients (mean age: 64.8 ± 11.7 years, females: 24.9%) who underwent transradial cardiac catheterization procedures for the purposes of invasive coronary angiography (ICA) (either undergoing PCIs or not) during the period of 2018–2021. The most common reason for ICA (43.3%) was the patient suffering from acute coronary syndromes (ACSs): 32.6% suffered from non-ST elevated ACS and 10.7% suffered from STEMI, with the remaining patients being referred for ICA because of stable coronary artery disease (CAD, 29%), valvular disease or heart failure, among others.

**Figure 1:**
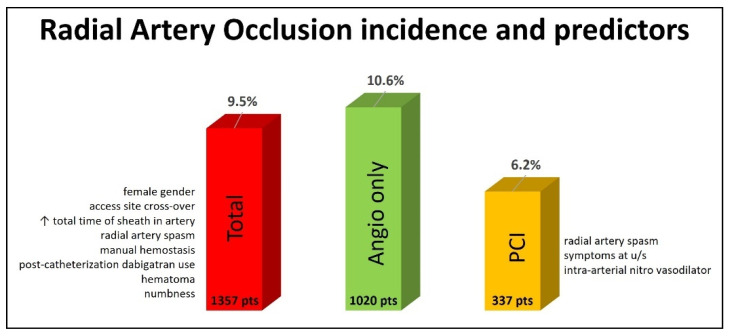
Incidence and predictors of radial artery occlusion at 24 h post-transradial cardiac catheterization.

Of the total population, 129 (9.5%) patients had RAO, as diagnosed by radial artery ultrasound 24 h after the procedure. Diagnostic angiography only was performed in 1020 patients, and 108 (10.6%) of them experienced RAO. A PCI was performed for 337 patients, and RAO was diagnosed in 21 (6.2%) of them (Figure 1).

The pre-procedural, procedural and post-procedural characteristics of our study population are presented in Table 1, Table 2 and Table 3, respectively, with comparisons between the RAO and radial artery patency (RAP) groups. Patients in the RAO group were older, received a lower dose of IV unfractionated heparin (UFH), were more likely to receive nitroglycerin as an intra-arterial vasodilator, underwent PCIs less frequently compared to the RAP group and had multiple puncture attempts. They also more frequently experienced radial artery spasms, access site cross-over (from the radial artery to another artery), manual hemostasis and symptoms at the forearm (pain and numbness) at the time the radial ultrasound was performed, while hemostasis was less successful.

Univariate and multivariate logistic regression models were used to identify any predictors of RAO in our population. The variables not associated with RAO occurrence are presented in Supplementary Table S1. The remaining predictors had potential significance in the univariate analyses (*p*-value < 0.10) and are described in Table 4.

A multivariate regression model was built to predict RAO occurrence and included the following variables: age, gender, smoking, heparin administration of more than 50 IU/kg, hematoma, successful hemostasis, hemostasis duration, initial sheath diameter, and total time of the sheath in the artery.

According to this multivariate adjustment process (Table 4), the following variables were independently linked with a higher risk of RAO:

(i) The female gender (aOR = 1.72 (1.05–2.83)), (ii) access site cross-over (aOR = 4.33 (1.02–18.39)), (iii) increased total time of the sheath in the artery (aOR = 1.01 (1.00–1.02)), (iv) radial artery spasms (aOR = 2.47 (1.40–4.36)), (v) the presence of hematomas (aOR = 2.28 (1.28–4.06)), (vi) post-catheterization dabigatran use (aOR = 5.15 (1.29–20.55)), (vii) manual hemostasis (aOR = 1.94 (1.01–3.72)) and (viii) numbness at radial artery ultrasound (aOR = 8.25 (1.70–40)).

Contrariwise, two variables were independently associated with increased odds for RAP: (i) PCI performance (aOR = 0.19 (0.06–0.63)) and (ii) a higher dosage of intravenous heparin per patient weight (aOR = 0.98 (0.96–0.99)), particularly a dosage of >50 IU/kg (aOR = 0.56 (0.31–1.00)).

Concerning our subgroup analysis including only the patients undergoing PCIs, of the 337 (24.8%) patients constituting this subgroup, RAO occurred in 21 of them (6.2%). We selected gender and heparin dosage per patient weight in kg for multivariate adjustments based on clinical relevance. After the multivariate adjustments, the following variables were independent predictors of RAO:

(i) Radial artery spasms (aOR = 4.48 (1.42–14.16)), (ii) the use of intra-arterial nitroglycerin as a vasodilator (aOR = 7.40 (1.67–32.79)) and (iii) the presence of symptoms at echo (aOR = 3.80 (1.46–9.87)), either pain (aOR = 2.93 (1.05–8.15)) or numbness (aOR = 4.66 (1.17–18.57)).

On the other hand, the use of an intra-arterial verapamil vasodilator (aOR = 0.17 (0.04–0.76)) was independently associated with a greater frequency of RAP.

## 4. Discussion

This study aimed at reporting the incidence of and identifying predictors of RAO in an all-comers European population that underwent a coronary catheterization procedure (angiography and/or a PCI) through conventional transradial access at the forearm. Our main finding was that RAO detected by radial artery ultrasound up to 24 h after the procedure had an incidence of 9.5% in the whole population, 10.6% in patients that underwent diagnostic angiography only and 6.2% in patients that underwent a PCI (Figure 1).

Regarding RAO predictors, eight factors were independently associated with higher risk of RAO, namely the following:

(A) Pre-procedural: (i) the female gender;
(B) Procedural: (ii) access site cross-over from the radial artery to another artery, (iii) an increased total time of the sheath remaining in the radial artery and (iv) radial artery spasms;
(C) Post-procedural: (v) the presence of hematomas, (vi) post-catheterization dabigatran use, (vii) manual hemostasis and (viii) numbness in the fingers of the punctured arm.

In the subgroup of PCI patients, only three variables were recognized as independent prognostic factors of RAO as follows:

(A) Procedural: (i) radial artery spasms, (ii) the use of intra-arterial nitroglycerin as a vasodilator;
(B) Post-procedural: (iii) the presence of symptoms, either pain or numbness.

In contrast, PCI performance and a higher dosage of intravenous heparin per patient weight (particularly a dosage of >50 IU/kg) in the whole group as well as the use of intra-arterial verapamil as a vasodilator in the PCI subgroup reduced the probability of RAO occurrence.

As stated above, RAO is the most frequent complication of forearm transradial catheterization, with an overall incidence ranging from 0.8 to 38% depending on several factors [13,14,15,16,17,18,19,20,21]. RAO incidence is also reported to be lower in patients undergoing PCIs (4.0–4.5%) versus those only having angiography performed (5.9–8.8%) [16,18]. The RAO incidence differs according to the time of evaluation. The RAO incidence is about 7.7% (0–17.4%) when evaluated early at 24 h, 9.5% between 24 h and 1 week and 5.56% 1 week post-procedure [16,18,46,47,48]. There is also a decrease in RAO incidence over time, probably due to the greater consideration and application of improved RAO preventive measures [19]. The RAO incidence also depends on the method of evaluation. Simple pulse palpation usually underestimates the incidence of RAO because of the palpable pulse of the radial artery, although it is occluded, due to reverse blood flow from anastomoses of the palmar arch. The oximetry/plethesmography test (reverse Barbeau test) offers better discrimination than simple pulse palpation; however, radial artery ultrasound remains the gold standard for RAO diagnosis [Rashid2016, Hahalis2017, Bernat2019]. Clinical pulse palpation significantly underestimates the incidence of RAO compared to ultrasound (4.4% vs. 10.5%, respectively) [42].

In this multicenter study, the overall RAO incidence (both the angiography and PCI groups) assessed for all patients at 24 h after the procedure with radial artery ultrasound was 9.5%, which is inside the range of 0–15.2% and slightly above the mean of 7.7% reported in the meta-analysis by Rashid et al. [16] (Figure 1). In the diagnostic angiography-only group, the RAO incidence was 10.6%, higher than the mean range of 5.9–8.8% reported in the meta-analyses by Rashid et al. and Hahalis et al., but lower than the 12% reported in the PROPHET and MEMORY studies, which included only diagnostic angiographies with 5-Fr sheaths [16,17,18,49]. Our study was not designed to test any specific method for RAO prevention with a strict protocol but to record the RAO incidence in an all-comers population. So, the angiography was performed either with 5-Fr or 6-Fr sheaths, intra-arterial vasodilators (nitroglycerin or verapamil) and a UFH dosage of 50 IU/kg or a standard of 5000 IU irrespective of the patient’s weight. The hemostasis method (device or manual) was based on the operator’s discretion. The RAO rate of 10.6% reflects all these variations.

In the PCI group, the RAO incidence was 6.2%, higher again than the mean range of 4.0–4.5% reported in the meta-analyses by Rashid et al. and Hahalis et al. but much lower than the extreme values of 11.3–32.9% reported in more recent studies [16,18,47,48,50,51,52] (Figure 1). We state again that this was an unselected population with no specialized equipment used (like slender guiding catheters), and all peri-procedural parameters were decided by the operator. However, the present study is one of the largest and most recent ones, including a wide spectrum of indications for diagnostic and interventional transradial procedures.

RAO develops as a result of thrombus formation in the radial artery due to mechanical and rheological factors. Punctures of the arterial wall, sheath insertion, catheter and wire introduction and manipulation, local micro-dissections and the compression of the artery during hemostasis or by hematoma formation all contribute to endothelial injury, reduced blood flow and local blood stasis, which, according to Virchow’s triad, initiate a coagulation cascade and facilitate thrombus formation [25,28,37,38,39,40,41,42,43]. Since RAO is recognized as the most frequent complication of transradial catheterization, researchers have been constantly trying to recognize factors that predispose patients to it and overcome them. For didactic reasons, they are categorized as follows:

(A) Pre-procedural (non-modifiable): age, the female gender, a low body mass index (BMI), diabetes mellitus, chronic kidney disease, peripheral arterial disease and smoking;
(B) Procedural (modifiable): previous ipsilateral radial artery punctures, inadequate periprocedural anticoagulation, the administration and type of intra-arterial vasodilators (sheath size, radial artery diameter and sheath-to-radial artery diameter ratio >1), repeated radial punctures, radial artery spasms, the procedural duration, aspirin use, statis use and glycoprotein inhibitor (GPI) use;
(C) Post-procedural (modifiable): occlusive (non-patent) or prolonged hemostasis.

However, studies keep on coming up with opposing evidence for many of these, probably reflecting differences in sample size, the characteristics of the population included and the methods applied in each of them [11,16,19,21,30].

In the present study, we tried to address this issue in a large sample of an unselected European, Caucasian population, coming from multiple tertiary hospitals’ catheterization labs. Regarding the pre-procedural risk factors for RAO, only age was found to be different between the RAO and RAP groups, with patients diagnosed with RAO being older (Table 1). No differences in gender, body habitus, medical history or indication for the procedure were detected. With respect to procedural predictors of RAO (Table 2), the RAO group received a lower dose of IV unfractionated heparin (UFH) and more nitroglycerin than verapamil as an intra-arterial vasodilator. The RAO patients also underwent fewer PCIs and more frequently had multiple puncture attempts, radial artery spasms and the need for access site cross-over (from the radial artery to another artery). There were no differences in the radial artery diameter or in the duration that the sheath remained in the artery. Post-procedurally, the RAO group received more manual hemostasis with less patency, which was less successful (more hematomas). The patients also experienced more symptoms (pain or numbness) at 24 h (Table 3).

The univariate analysis (Table 4) showed that pre-procedural factors predisposing patients to RAO were the female gender, an older age and smoking. Procedural univariate RAO predictors were multiple punctures of the radial artery, an increased total time of the sheath remaining in the radial artery, radial artery spasms, the need for access site cross-over (from the radial artery to another artery) and the use of nitroglycerin as an intraarterial vasodilator. Post-procedural RAO predictors were the application of manual hemostasis, dabigatran use and the emergence of symptoms at the forearm (pain or numbness). On the contrary, factors favoring RAP were the use of a smaller sheath diameter, verapamil as an intra-arterial vasodilator, a PCI being performed, more UFH administered and the achievement of successful hemostasis.

However, in the multivariate logistic regression analysis, independent predictors of RAO were as follows:

- The female gender: The female gender has been described as predisposing patients to RAO in several studies. It is assumed that their lower BMI and their decreased radial artery diameter, especially when combined with the use of large bore sheaths, predispose these patients to more spasms and radial artery injury and subsequently to RAO. However, this is not a consistent finding among all studies. In our study, being female was a predictor for increased RAO only in the angiography group, while it lost its significance in the PCI subgroup, indicating that procedural and post-procedural factors are probably more important when proceeding to PCIs [11,16,18,19,30,53].
- Access site cross-over from the radial artery to another artery: The need for cross-over from the radial artery to another artery usually implies that either the artery was punctured multiple times with no successful sheath insertion or that after the sheath insertion, a complication occurred, like a radial artery spasm or injury to the radial artery, and the procedure could not be completed through the initial access site. Moreover, loops of the radial artery at the forearm or subclavian artery tortuosity may lead to cross-over either due to spasms or due to the inability to cannulate the coronary arteries. In each case, however, many catheters, wires and manipulation maneuvers are usually applied. In all of the above cases, the common denominator is the increased injury to the radial artery wall and, as stated in the pathophysiology explanation, this induces RAO. Not many studies have evaluated this, probably because they focus on other endpoints, like multiple punctures and spasms (which, as described, can result in access site cross-over) [11,16,18,19,25,28,30,37,38,39,40,41,42,43]. In the present study, patients were included only if they had a successful insertion of at least one radial artery sheath, so sheath-related arterial wall injury (and not only puncture/needle injury) was a case for all our patients. However, we did not systematically record the cause of cross-over (spasms, radial or subclavian tortuosity or other reasons), so we cannot provide more details about that. Access site cross-over was a significant RAO predictor only for the whole group and not for the PCI subgroup [11,16,18,19,30].
- An increased total time of the sheath remaining in the radial artery: The radial sheath, apart from the injuries it causes to the arterial wall during its insertion, also reduces blood flow locally and promotes blood stasis and thrombus formation. However, there are studies with opposing evidence on whether a longer or shorter procedure duration promotes RAO (expressed usually as the procedural time) [11,16,18,19,25,28,30,37,38,39,40,41,42,43]. We recorded the time from sheath insertion until sheath removal and found that this was a borderline significant predisposing factor for RAO in the whole population but not in the PCI subgroup.
- Radial artery spasm: Spasms of the radial artery are a well-recognized factor that increases RAO rates and are implicated as such in many studies, although they were not proven as a main factor in the meta-analyses by Rashid et al. and Hahalis et al. [11,16,18,19,30]. However, spasm prevention is recommended, and relevant measures should be applied, mainly by the use of vasodilators [19]. Vascular spasms likely indicate a response of the artery to injury or friction with the equipment, subsequently reducing blood flow and promoting RAO. In the present study, a radial artery spasm was recognized as an independent RAO predictor both for the whole population and for the PCI subgroup.
- Radial artery vasodilators: Nitroglycerin is a vasodilating agent and is traditionally used intra-arterially during transradial catheterization to prevent radial artery spasms, usually at a dose of 200–500 μg. However, in the latest randomized study and the latest meta-analysis, intra-arterial nitroglycerin did not show any advantage in preventing radial artery spasms or RAO [54,55]. Only subcutaneous nitroglycerin was found to prevent radial spasms or RAO [55]. Verapamil, a calcium channel blocker, is also used intra-arterially as a vasodilator during transradial catheterization, at a dose of 2.5–5 mg. These two agents can be also combined and administered simultaneously in transradial procedures [56]. Verapamil only or verapamil in combination with nitroglycerin is probably more effective in preventing radial spasms and RAO than nitroglycerin alone [56,57]. In the present study, when nitroglycerin was selected from the operator as a vasodilator, it was administered intra-arterially, just after the sheath placement at a dosage of 200 μg. When verapamil was selected, it was also administered intra-arterially just after the sheath insertion at a dosage of 2.5 mg. The multivariate analysis showed that vasodilators had no effect on RAO for the whole cohort, but for the PCI subgroup, nitroglycerin compared to verapamil increased the odds of RAO, while verapamil seems to have a favorable effect in preventing RAO.
- The presence of hematoma: Hematoma formation in the forearm represents another complication of transradial catheterization. It is usually due to radial or brachial artery perforation and indicates severe radial artery trauma from one side, while the blood that accumulates in the forearm region compresses from outside of the radial artery on the other side. Moreover, in order to manage and minimize the extent of hematomas, additional external direct pressure on the forearm usually with a sphygmomanometer and a pressure bandage has to be applied. All these events promote blood stasis and may predispose patients to RAO [11,21,30,44]. In the present study, the presence of any hematoma in the forearm according to the EASy classification was a predictor for RAO in the whole patient group but not in the PCI subgroup.
- Post-catheterization dabigatran use: Dabigatran is a non-vitamin K antagonist oral anticoagulant (NOAC) and acts as a direct thrombin (factor II) inhibitor in the coagulation cascade. It is indicated for thromboembolism prevention in patients with atrial fibrillation and for the treatment of deep vein thrombosis and pulmonary embolism [58]. It would be better like this: “Although very effective for the prevention and treatment of thrombi in the venous circulation (lower shear stress and velocity) and the left atrial appendage, when it comes to the arterial circulation and when devices are implicated, there are some observations that may need attention. In the RE-DUAL PCI study, in which dabigatran was used (instead of warfarin) as part of a dual antithrombotic regimen in combination with a clopidogrel or ticagrelor in patients with atrial fibrillation undergoing PCIs, the dabigatran groups had numerically (although not statistically significant) more myocardial infarctions [59]. Dabigatran also showed increased thromboembolic events in patients with mechanical heart valves, left ventricular assist devices and when used periprocedurally during radiofrequency ablation for atrial fibrillation [60,61,62]. The above data may indicate that dabigatran is not very effective in preventing arterial thrombi or may increase thrombogenicity when devices and catheters are inserted in the arterial circulation, as is the case in coronary angiography. In contrast, rivaroxaban, another NOAC acting as a direct factor X inhibitor, showed more promising results in preventing arterial or device-related thrombi [63,64,65]. That is the reason we checked dabigatran and found it to be an independent predictor of RAO for the whole group but not for the PCI subgroup. We recognize that this is a finding not described before and may not look reasonable at first sight, but it could be further investigated in larger samples of patients receiving dabigatran peri-procedurally during transradial catheterization.
- Manual hemostasis: Instead of placing a device, hemostasis can be achieved by direct pressure on the puncture site after sheath removal by the operator or nurse. Despite a shorter hemostasis time (which could be beneficial for RAO prevention), manual compression depends on the person applying the pressure, making it very variable and not reproducible [66]. Probably, especially during the first minutes after sheath removal, a very high level of pressure is applied in order to avoid bleeding, and this deteriorates blood flow in the already traumatized radial artery. When compared to device hemostasis in a randomized study, manual hemostasis had a similar RAO incidence [17]. In the present study, manual hemostasis was applied only after diagnostic coronary angiography and was found to be an independent predictor of RAO.
- Presence of symptoms (pain in the forearm or numbness in the fingers of the punctured arm): RAO may be symptomatic in up to 58.8% of patients, presenting as pain in the forearm, numbness/paresthesia, paresis or acute ischemia [20,22,23,24,25,26]. Forearm nerve injury may happen either directly, during puncture or during device hemostasis, or indirectly due to radial artery injury, spasms or hematomas and subsequent nerve ischemia. The presence of symptoms probably indicates a higher degree of radial artery injury, further predisposing patients to RAO. In this study, RAO was symptomatic in one third of patients, expressed mainly as pain in the forearm. Pain was an independent predictor of RAO in the PCI subgroup, while numbness was an RAO predictor both for the whole group and the PCI subgroup.

In this study, PCI performance and a higher dosage of intravenous heparin per patient weight (particularly a dosage of >50 IU/kg) in the whole group reduced the probability of RAO occurrence, as described in most studies and clearly depicted in meta-analyses [11,16,18,19,30]. The fact that higher doses of UFH have a consistent favorable effect on reducing RAO indirectly supports the thrombus formation theory in RAO pathophysiology. Other non-consistently described RAO predictors, like age, smoking, a lower glomerular filtration rate, multiple puncture attempts, sheath size, contrast volume, patent hemostasis and hemostasis duration, were not confirmed in the present study. A sheath-to-radial artery diameter ratio > one, a well-recognized RAO predictor, was also not an independent factor predisposing patients to RAO in this study. This is probably due to the small percentage (less than 2%) of sheaths > 6-Fr that were included in our study.

Our study is one of the largest multicenter studies dealing with RAO incidence and predictors in transradial angiography and PCI and in sample sizes of more than 1000 patients [48,51,52,53]. The reported RAO incidence of 9.5% at 24 h is lower than that in all other studies (11.3–17.4%), except for that of Schlosser et al. (4.6%) [48,51,52,53]. In accordance with these studies, the RAO predictors found in the present study are the female gender [48,52,53] and the presence of forearm hematomas [51].

Several measures to prevent RAO have been proposed, like reducing sheath/catheter size, adequate procedural anticoagulation, patent hemostasis, a short hemostasis time [19] and ultrasound-guided radial artery punctures to reduce the number of puncture attempts [67]. Another important preventive measure that has recently evolved is the use of the distal radial artery, punctured at the anatomical snuffbox, as an access site for coronary catheterization. Distal transradial access compared to traditional forearm transradial access has shown improved RAO rates in several randomized studies [68,69,70,71,72]. In case RAO happens, there are also options to try to re-establish patency either conservatively (anticoagulation or ulnar artery compression) or invasively with balloon angioplasty [11,73]. A detailed description of these is out of the scope of the current manuscript.

## 5. Limitations

This was an observational study with no strict protocol, and all periprocedural decisions were at each operator’s discretion (concerning anticoagulation, sheath size, vasodilators, hemostasis method and duration). We also did not conduct an ultrasound doppler examination for the radial artery before the procedure, which is important in understanding its anatomy, and could aid significantly in ensuring the safety of the procedure, avoiding possible complications, while also helping us understand and stratify the predictors of RAO. Finally, the reason for access site cross-over (radial artery spasms or tortuosity/loops) was not systematically recorded.

## 6. Conclusions

The incidence of RAO in an unselected, all-comers European population after transradial coronary catheterization for angiography and/or PCI is similar to that reported in the international literature. In PCI patients, RAO had a lower incidence than in angiography-only patients. The female gender, radial artery trauma (access site cross-over from the radial artery to another artery, an increased total time of the sheath remaining in the radial artery, radial artery spasms, the presence of hematoma and patient symptoms) and manual hemostasis are the strongest predictors of RAO. In contrast, for PCI patients, a higher dosage of intravenous heparin per patient weight (particularly a dosage of >50 IU/kg) and intra-arterial verapamil as a vasodilator reduce the probability of RAO occurrence.

## Figures and Tables

**Table 1:** Pre-procedural characteristics based on the presence of radial artery occlusion.

Variable	Radial Artery Occlusion (*n* = 129)	Radial Artery Patency (*n* = 1222)	*p*-Value
Age (years) ^#^	66 (21)	65 (16)	***p* = 0.03**
Female gender	40 (31%)	297 (24.3%)	*p* = 0.09
BMI (kg/m^2^) ^#^	29 (7.3)	29 (5.5)	*p* = 0.28
BSA (m^2^) ^#^	1.9 (0.2)	2 (0.3)	*p* = 0.77
GFR (mL/min/1.73 m^2^) ^#^	89 (37)	85 (47)	*p* = 0.11
Platelets (10^9^/L) ^#^	241 (98)	222 (79)	*p* = 0.33
LVEF (%) ^#^	55 (27.5)	55 (15)	*p* = 0.53
CHA_2_DS_2_-VASc ^#^	2 (3)	3 (3)	*p* = 0.28
Hemoglobin (g/dL) ^#^	13.8 (2.5)	13.9 (2)	*p* = 0.59
INR ^#^	0.97 (0.1)	0.99 (0.1)	*p* = 0.57
Smoking	63 (49.6%)	482 (41.1%)	*p* = 0.07
ACS presentation	50 (44.2%)	451 (41.9%)	*p* = 0.63
STEMI presentation	12 (10.6%)	118 (11%)	*p* = 0.91
Stable CAD presentation	34 (30.1%)	317 (29.5%)	*p* = 0.89
Prior CAD	32 (25.2%)	339 (33.7%)	*p* = 0.20
Prior PCI	14 (20.6%)	155 (27%)	*p* = 0.26
PAD	2 (3%)	33 (5%)	*p* = 0.47
Stroke	3 (4.5%)	23 (3.5%)	*p* = 0.67
Hypertension	79 (62.2%)	713 (60.8%)	*p* = 0.76
Diabetes mellitus	43 (33.9%)	362 (30.9%)	*p* = 0.49
Dyslipidemia	56 (44.1%)	520 (44.3%)	*p* = 0.96
Family CAD	20 (15.7%)	166 (14%)	*p* = 0.59
Atrial fibrilation	10 (14.9%)	87 (13.1%)	*p* = 0.67
Heart failure	15 (22.1%)	140 (21%)	*p* = 0.84
CKD	3 (4.4%)	32 (5%)	*p* = 0.83
Antiplatelets pre	81 (70.4%)	751 (67.8%)	*p* = 0.56
Aspirin pre	71 (61.7%)	660 (59.6%)	*p* = 0.65
Clopidogrel pre	45 (39.1%)	411 (37.1%)	*p* = 0.67
Ticagrelor pre	11 (9.6%)	81 (7.3%)	*p* = 0.38
OACs pre	16 (23.2%)	175 (25.5%)	*p* = 0.67
LMWH pre	8 (11.6%)	103 (15%)	*p* = 0.44
NOACs pre	7 (10.4%)	65 (9.5%)	*p* = 0.80
Thrombolysis	2 (2.9%)	15 (2.2%)	*p* = 0.70
Preloading asp+clop	6 (8.7%)	48 (7%)	*p* = 0.60
Preloading asp+tic	8 (11.6%)	63 (9.2%)	*p* = 0.52

^#^ According to the Kolmogorov–Smirnov test, all of the above variables did not follow normal distribution, and, therefore, we used the Mann–Whitney U test for the comparison of their values based on the presence of radial artery occlusion. Abbreviations: BMI: Body mass index, BSA: Body surface area, INR: International normalized ratio, GFR: Glomerular filtration rate, PCI: Percutaneous coronary intervention, LVEF: Left ventricular ejection fraction, IV: intravenous, UFH: Unfractionated heparin, ACS: Acute coronary syndrome, STEMI: ST-elevation myocardial infarction, CAD: Coronary artery disease, PCI: Percutaneous coronary intervention, PAD: Peripheral arterial disease, CKD: Chronic kidney disease, OAC: Oral anticoagulant, LMWH: Low-molecular-weight heparin, NOAC: Non-vitamin K antagonist oral anticoagulant and UFH: Unfractionated heparin. Statistical significant *p* values are presented in bold.

**Table 2:** Procedural characteristics based on the presence of radial artery occlusion.

Variable	Radial Artery Occlusion (*n* = 129)	Radial Artery Patency (*n* = 1222)	*p*-Value
Left arm	5 (3.9%)	57 (4.8%)	*p* = 0.81
IV UFH > 50 IU/kg	46 (35.7%)	554 (45.3%)	***p* = 0.04**
IV UFH (IU) ^#^	4000 (1400)	4500 (1075)	*p* = 0.07
Radial artery spasm	16 (15.7%)	62 (7.2%)	***p* < 0.01**
Access site cross-over	7 (10.6%)	18 (2.9%)	***p* < 0.01**
Radial artery diameter at puncture site (mm) ^#^	2.7 (0.6)	3 (0.7)	*p* = 0.25
Final sheath diameter 5 Fr	86 (67.2%)	671 (57.1%)	***p* = 0.03**
Final sheath diameter 6 Fr	40 (31.3%)	491 (41.8%)	***p* = 0.02**
Final sheath diameter 7 Fr	2 (1.6%)	13 (1.1%)	*p* = 0.65
Sheath/artery ratio ^#^	0.6 (0.2)	0.6 (0.1)	***p* = 0.05**
Intra-arterial nitroglycerin as vasodilator	81 (70.4%)	623 (56.3%)	***p* < 0.01**
Intra-arterial verapamil vasodilator	31 (27%)	412 (37.3%)	***p* = 0.03**
Obstructive CAD identified	43 (62.3%)	476 (70%)	*p* = 0.19
Multivessel CAD identified	33 (25.6%)	320 (26.2%)	*p* = 0.88
PCI performed	21 (16.3%)	314 (25.8%)	***p* = 0.02**
>one puncture attempt	30 (26.3%)	168 (18.2%)	***p* = 0.04**
Total time of sheath in artery (min) ^#^	30 (30)	25 (22)	*p* = 0.46
Radiation time (min) ^#^	4 (4)	7 (8)	*p* = 0.45
Contrast volume (ml) ^#^	100 (25)	100 (70)	*p* = 0.30
Radiation (mGy) ^#^	147 (418)	496 (652)	*p* = 0.61

^#^ According to the Kolmogorov–Smirnov test, all of the above variables did not follow normal distribution, and, therefore, we used the Mann–Whitney U test for the comparison of their values based on the presence of radial artery occlusion. Abbreviations: BMI: Body mass index, BSA: Body surface area, INR: International normalized ratio, GFR: Glomerular filtration rate, PCI: Percutaneous coronary intervention, LVEF: Left ventricular ejection fraction, IV: intravenous, UFH: Unfractionated heparin, ACS: Acute coronary syndrome, STEMI: ST-elevation myocardial infarction, CAD: Coronary artery disease, PCI: Percutaneous coronary intervention, PAD: Peripheral arterial disease, CKD: Chronic kidney disease, OAC: Oral anticoagulant, LMWH: Low-molecular-weight heparin, NOAC: Non-vitamin K antagonist oral anticoagulant and UFH: Unfractionated heparin. Statistical significant *p* values are presented in bold.

**Table 3:** Post-procedural characteristics based on the presence of radial artery occlusion.

Variable	Radial Artery Occlusion (*n* = 129)	Radial Artery Patency (*n* = 1222)	*p*-Value
Hemostasis duration (min) ^#^	75 (167)	60 (94)	*p* = 0.50
Successful hemostasis	108 (84.4%)	1080 (92.2%)	***p* < 0.01**
Manual hemostasis	41 (31.8%)	285 (23.6%)	***p* = 0.04**
Patent hemostasis	49 (62%)	572 (87.5%)	***p* < 0.01**
Hematoma	37 (28.9%)	160 (13.5%)	***p* < 0.01**
Hemorrhage	7 (5.5%)	51 (4.3%)	*p* = 0.52
Palpable pulse	22 (17.1%)	1177 (96.3%)	***p* < 0.01**
Symptoms at radial ultrasound	22 (31.9%)	74 (10.8%)	***p* < 0.01**
Pain	19 (27.5%)	64 (9.3%)	***p* < 0.01**
Numbness	6 (8.7%)	14 (2%)	***p* < 0.01**
Antithrombotics post-cath	60 (87%)	579 (90%)	*p* = 0.42
Post-aspirin	53 (76.8%)	500 (77.6%)	*p* = 0.88
Post-clopidogrel	23 (33.3%)	250 (38.8%)	*p* = 0.37
Post-ticagrelol	17 (24.6%)	188 (29.2%)	*p* = 0.43
Post-prasugrel	0 (0%)	5 (0.8%)	*p* = 0.46
Post-acenocoumarol	2 (2.9%)	6 (0.9%)	*p* = 0.14
Post-NOACs	9 (13%)	61 (9.5%)	*p* = 0.34

^#^ According to the Kolmogorov–Smirnov test, all of the above variables did not follow normal distribution, and, therefore, we used the Mann–Whitney U test for the comparison of their values based on the presence of radial artery occlusion. Abbreviations: BMI: Body mass index, BSA: Body surface area, INR: International normalized ratio, GFR: Glomerular filtration rate, PCI: Percutaneous coronary intervention, LVEF: Left ventricular ejection fraction, IV: intravenous, UFH: Unfractionated heparin, ACS: Acute coronary syndrome, STEMI: ST-elevation myocardial infarction, CAD: Coronary artery disease, PCI: Percutaneous coronary intervention, PAD: Peripheral arterial disease, CKD: Chronic kidney disease, OAC: Oral anticoagulant, LMWH: Low-molecular-weight heparin, NOAC: Non-vitamin K antagonist oral anticoagulant and UFH: Unfractionated heparin. Statistical significant *p* values are presented in bold.

**Table 4:** Univariate analysis with *p*-value < 0.10 and multivariate analyses yielding independently significant predictors of radial artery occlusion.

	Univariate	Multivariate Whole Group	Multivariate Only for PCI Subgroup
**Predictor**	**OR (95% CI), *p*-Value**	aOR * (95% CI), *p*-Value	aOR ** (95% CI), *p*-Value
Pre-procedural			
Female gender	OR = 1.40 (0.94–2.08), *p* = 0.10	**aOR = 1.72 (1.05–2.83), *p* = 0.03**	aOR = 1.16 (0.37–3.70), *p* = 0.80
Age	OR = 0.98 (0.97–1.00), *p* = 0.01	aOR = 0.99 (0.97–1.01), *p* = 0.14	aOR = 0.98 (0.94–1.02), *p* = 0.39
Smoking	OR = 1.41 (0.98–2.04), *p* = 0.07	aOR = 1.32 (0.81–2.15), *p* = 0.26	aOR = 1.74 (0.66–4.59), *p* = 0.26
Procedural			
>one puncture attempt	OR = 1.60, 1.02–2.51, *p* = 0.04	aOR = 1.53 (0.91–2.57), *p* = 0.11	aOR = 0.39 (0.05–3.15), *p* = 0.38
Sheath diameter (Fr)	OR = 0.67 (0.45–0.99), *p* = 0.05	aOR = 0.73 (0.36–1.49), *p* = 0.39	aOR = 0.69 (0.19–2.52), *p* = 0.57
Total time of sheath in artery	OR = 1.00 (0.99–1.01), *p* = 0.82	**aOR = 1.01 (1.00–1.02), *p* = 0.05**	aOR = 1.01 (0.99–1.02), *p* = 0.35
Radial spasm	OR = 2.73 (1.72–4.34), *p* < 0.01	**aOR = 2.47 (1.40–4.36), *p* < 0.01**	**aOR = 4.48 (1.42–14.16), *p* = 0.01**
Cross-over	OR = 4.01 (1.61–9.99), *p* < 0.01	**aOR = 4.33 (1.02–18.39), *p* = 0.05**	aOR = 2.16 (0.25–19.03), *p* = 0.49
Intra-arterial nitroglycerin as vasodilator	OR = 1.85 (1.22–2.81), *p* < 0.01	aOR = 1.06 (0.61–1.86), *p* = 0.83	**aOR = 7.40 (1.67–32.79), *p* < 0.01**
Intra-arterial verapamil vasodilator	OR = 0.62 (0.41–0.96), *p* = 0.03	aOR = 0.98 (0.56–1.72), *p* = 0.94	**aOR = 0.17 (0.04–0.76), *p* = 0.02**
PCI performed	OR = 0.56 (0.34–0.91), *p* = 0.02	**aOR = 0.19 (0.06–0.63), *p* < 0.01**	n.a.
IV UFH (IU per kg)	OR = 0.99 (0.99–1.00), *p* = 0.04	**aOR = 0.98 (0.96–0.99), *p* < 0.01**	aOR = 1.00 (0.99–1.01), *p* = 0.94
IV UFH > 50 IU/per kg	OR = 0.67 (0.46–0.98), *p* = 0.04	**aOR = 0.56 (0.31–1.00), *p* = 0.05**	aOR = 4.15 (0.55–31.61), *p* = 0.17
Post-procedural			
Successful hemostasis	OR = 0.46 (0.27–0.77), *p* < 0.01	aOR = 0.81 (0.31–2.14), *p* = 0.68	aOR = 0.66 (0.24–1.82), *p* = 0.42
Manual hemostasis	OR = 1.51 (1.02–2.24), *p* = 0.04	**aOR = 1.94 (1.01–3.72), *p* = 0.05**	n.a.
Hematoma	OR = 2.62 (1.72–3.97), *p* < 0.01	**aOR = 2.28 (1.28–4.06), *p* < 0.01**	aOR = 1.82 (0.65–5.06), *p* = 0.25
Post-cath dabigatran	OR = 3.52 (1.23–10.08), *p* = 0.02	**aOR = 5.15 (1.29–20.55), *p* = 0.02**	aOR = 3.61 (0.37–34.84), *p* = 0.27
Symptoms at radial artery ultrasound	OR = 3.89 (2.22–6.81), *p* < 0.01	aOR = 2.10 (0.79–5.55), *p* = 0.14	**aOR = 3.80 (1.46–9.87), *p* < 0.01**
Pain at radial artery ultrasound	OR = 3.71 (2.06–6.67), *p* < 0.01	aOR = 1.64 (0.58–4.66), *p* = 0.35	**aOR = 2.93 (1.05–8.15), *p* = 0.04**
Numbness at radial artery ultrasound	OR = 4.59 (1.70–12.35), *p* < 0.01	**aOR = 8.25 (1.70–40), *p* < 0.01**	**aOR = 4.66 (1.17–18.57), *p* = 0.03**

* All multivariate regression models were adjusted for the following: age, gender, smoking, UFH > 50 IU/kg, hematoma, successful hemostasis, hemostasis duration, initial sheath diameter, time of hemostasis and total time of sheath in artery. ** In the PCI subgroup population, all multivariate regression models were adjusted for the following: gender and heparin dosage per patient weight in kg. Abbreviations: PCI: Percutaneous coronary intervention, IV: Intravenous, UFH: Unfractionated heparin, n.a.: not applicable. Statistical significant *p* values are presented in bold.

## Data Availability

The raw data supporting the conclusions of this article will be made available by the authors upon request.

## Supplementary Materials

The following supporting information can be downloaded at: https://www.mdpi.com/article/10.3390/jcm13113276/s1, Table S1: Univariate analyses yielding *p*-value > 0.10 (non-significant).
