# Supplementary material for: Incidence and Prognostic Factors of Radial Artery Occlusion in Transradial Coronary Catheterization
Source: J Clin Med. 2024 Jun 1;13(11):3276. doi: 10.3390/jcm13113276 (PMC11173088; doi:10.3390/jcm13113276)
Supplement: Supplementary file 1 [file jcm-13-03276-s001.zip › jcm-2974994-supplementary.pdf]

Journal of
Clinical Medicine

MDPI

Supplementary Table S1. Univariate analyses yielding *p*-value >0.10 (non-significant).

Predictor	OR, 95% CI	*p*-value
Number of diseased vessels	0.88,0.68-1.12	*p*=0.30
Hemorrhage	1.30, 0.58-2.93	*p*=0.53
Age >=70	0.76, 0.52-1.12	*p*=0.17
Age >=80	0.70, 0.36-1.36	*p*=0.29
BMI	1.02, 0.98-1.06	*p*=0.37
BMI >=20	1.95, 0.26-14.74	*p*=0.52
BMI >=25	1.16, 0.71-1.87	*p*=0.56
BMI>=30	1.29, 0.88-1.88	*p*=0.19
BMI >=35	1.23, 0.70-2.14	*p*=0.47
BMI>=40	0.82, 0.25-2.70	*p*=0.74
BSA	1.02, 0.44-2.36	*p*=0.97
BSA >=1.6	0.92, 0.41-2.05	*p*=0.84
BSA >=1.7	0.73, 0.42-1.27	*p*=0.26
BSA >=1.8	1.06, 0.67-1.69	*p*=0.79
BSA >=1.9	1.07, 0.73-1.58	*p*=0.72
Hemoglobin	0.99, 0.90-1.10	*p*=0.92
Platelets	1.00, 0.99-1.00	*p*=0.48
INR	0.74, 0.15-3.63	*p*=0.71
GFR	1.00, 1.00-1.01	*p*=0.16
Hx CAD	0.76, 0.50-1.16	*p*=0.20
Prior PCI	0.70, 0.38-1.30	*p*=0.26
Hypertension	1.06, 0.73-1.55	*p*=0.76
Diabetes Mellitus	1.15, 0.78-1.69	*p*=0.49
Dyslipidemia	0.99, 0.69-1.43	*p*=0.96
Family CAD	1.15, 0.69-1.91	*p*=0.59
Atrial Fibrilation	1.17, 0.58-2.37	*p*=0.67
PAD	0.59, 0.14-2.52	*p*=0.48
Stroke	1.31, 0.38- 4.48	*p*=0.67
Heart Failure	1.07, 0.58-1.95	*p*=0.84
LVEF	0.99, 0.96-1.02	*p*=0.35
LVEF<40	0.98, 0.47-2.05	*p*=0.96
LVEF<50	1.09, 0.62-1.91	*p*=0.78
CKD	0.88, 0.26-2.94	*p*=0.83
Antithrombotics pre	0.68, 0.37-1.22	*p*=0.20
Antiplatelets pre	1.13, 0.74-1.72	*p*=0.56
Aspirin pre	1.10, 0.74-1.63	*p*=0.65
Clopidogrel pre	1.09, 0.74-1.62	*p*=0.67
Ticagrelor pre	1.34, 0.69-2.60	*p*=0.38
OACs pre	0.88, 0.49-1.58	*p*=0.67
LMWH pre	0.74, 0.34-1.59	*p*=0.44
NOACs pre	1.11, 0.49-2.53	*p*=0.80
Thrombolysis	1.33, 0.30-5.96	*p*=0.71
Preloading aspirin+clopidogrel	1.27, 0.52-3.07	*p*=0.61
Preloading aspirin+ticagrelor	1.29, 0.59-2.82	*p*=0.52
Left arm vs right arm	0.76, 0.31-1.88	*p*=0.55
Puncture attempts	1.10, 0.94-1.28	*p*=0.24
Obstructive CAD identified	0.71, 0.42-1.19	*p*=0.19

Multivessel CAD identified	0.97, 0.64-1.47	*p*=0.88
ACS presentation	1.10, 0.74-1.63	*p*=0.63
STEMI presentation	0.97, 0.52-1.81	*p*=0.91
Stable CAD presentation	1.03, 0.68-1.57	*p*=0.89
Total time of sheath in artery	1.00, 0.99-1.01	*p*=0.82
Radiation time	1.01, 0.99-1.03	*p*=0.54
Contrast volume	1.00, 1.00-1.00	*p*=0.31
Radiation (mGy)	1.00, 1.00-1.00	*p*=0.44
Hemostasis duration	1.00, 1.00-1.00	*p*=0.64
Hemostasis >1 hour	0.81, 0.53-1.24	*p*=0.33
Hemostasis >2 hours	0.88, 0.58-1.35	*p*=0.57
Hemostasis >2.5 hours	1.36, 0.62-2.95	*p*=0.44
Patent hemostasis	1.45, 0.79-2.65	*p*=0.23
Radial artery diameter	0.82, 0.55-1.22	*p*=0.33
Ratio of sheath diameter to artery diameter	2.93, 0.63-13.56	*p*=0.17
Heparin IV >5000IU	0.71, 0.43-1.17	*p*=0.17
Heparin IV >10000 IU	1.10, 0.53-2.30	*p*=0.80
Sheath exchange	0.94, 0.33-2.67	*p*=0.91
Post-cath antithrombotics	0.74, 0.35-1.56	*p*=0.42
Post-cath aspirin	0.96, 0.53-1.72	*p*=0.88
Post-cath clopidogrel	0.79, 0.47-1.33	*p*=0.37
Post-cath ticagrelol	0.79, 0.45-1.41	*p*=0.43
Post-cath acenocoumarol	3.17, 0.63-16.01	*p*=0.16
Post-cath NOACs	1.43, 0.68-3.03	*p*=0.35
Post-cath rivaroxaban	1.17, 0.34-4.00	*p*=0.80
Post-cath apixaban	0.40, 0.05-2.99	*p*=0.37

Abbreviations: BMI: Body mass index, BSA: Body Surface Area, INR: International Normalized Ratio, GFR: Glomerular filtration rate, CAD: Coronary artery disease, PCI: Percutaneous coronary intervention, PAD: Peripheral Arterial Disease, LVEF: Left Ventricular Ejection Fraction, CKD: Chronic Kidney Disease, OAC: Oral Anticoagulant, LMWH: Low-Molecular-Weight Heparin, IV: intravenous, NOAC: Novel Oral Anticoagulant, ACS: Acute Coronary Syndrome, STEMI: ST-elevation myocardial infarction.
